# Supplementary material for: Treatment outcomes in HIV infected patients older than 50 years attending an HIV clinic in Harare, Zimbabwe: A cohort study
Source: PLoS One. 2021 Jun 9;16(6):e0253000. doi: 10.1371/journal.pone.0253000 (PMC8189507; doi:10.1371/journal.pone.0253000)
Supplement: Attachment — Submitted filename: Response to Reviewers.pdf [file pone.0253000.s004.pdf]

Newlands Clinic

PLOS ONE
Editorial Office
1160 Battery Street,
Suite 225,
San Francisco, CA 94111

20 May 2021

Case Number: 07083098

ref:_00DU0Ifis._5004P1TskEb:ref

Dear Dr Hyang Nina Kim,

Thank you for the follow-up response to our re-submission. Below are responses to the two issues raised in the email dated 18 May 2021:

Comment 1

Please address the reviewer's comment re line 64 (formerly line 57) that discusses accelerated immune senescence and disease progression in the elderly. The reviewer point outs: "increased risk of opportunistic infections may not be true in the setting of viral suppression; please provide a reference and modify this comment as needed."

Response

We previously revised the original sentence suggesting increased risk of opportunistic infections (OI) as suggested by the reviewer and removed the OI connotation. The revised sentence reads as follows, "Management of OPLHIV requires a multidisciplinary team approach, taking into account the onset of non-communicable diseases, premature ageing, and an increased risk of drug-to-drug interactions and drug toxicities due to polypharmacy". To improve clarity, we have revised the preceding sentence as well which now reads, "Compounding the age-related thymic contraction, HIV infection affects both B- and T-cell function leading to accelerated immunosenescence and consequently increased risk of non-AIDS related, age associated comorbidities in elderly patients", with the relevant references added.

Comment 2

Table 4 requires further revision to remove reference to WHO staging in favor of baseline CD4 cell count which is more objectively assessed than the former. There also remains a footnote re WHO staging that will need to be removed.

Response

Newlands Clinic
56 Enterprise Road, Harare, Zimbabwe
Phone +263 (0)4 776433
info@newlandsclinic.org.zw

We have removed the row with WHO stage from Table 4 and indicated the unadjusted hazard ratio in the text. We have also corrected the Table 4 footnote which now reads, “Baseline WHO clinical stage was omitted from multivariable analysis due to collinearity with baseline CD4 T-cell count.”

We hope that these revisions adequately resolve the outstanding issues.

Sincerely,

Tinei Shamu
