# Supplementary material for: Treatment outcomes in HIV infected patients older than 50 years attending an HIV clinic in Harare, Zimbabwe: A cohort study
Source: PLoS One. 2021 Jun 9;16(6):e0253000. doi: 10.1371/journal.pone.0253000 (PMC8189507; doi:10.1371/journal.pone.0253000)
Supplement: Attachment — Submitted filename: PONE_review_20210310.docx [file pone.0253000.s002.docx]

PloS One Review

This cohort study of 420 patients who are living with HIV and over 50 years old presenting for care and started ART at a clinic in Harare, Zimbabwe over a 16 year period. This cohort demonstrates a high mortality rate (21%) despite high rates of viral suppression (94%). High mortality correlated with delayed initiation of ART (WHO stage 3-4 at initiation) (aHR=3.0) and male sex (aHR=2.0) as well as indicators of poverty (unemployment) and being unmarried. I would recommend revising the analysis to include baseline CD4 count rather than WHO stage as a more objective measure of immunosuppression at initiation of ART. This manuscript is well written aside from some minor grammatical errors (e.g., inconsistent use of oxford comma) and includes interesting data that indicate the importance of considering gender and socioeconomic status when considering management of persons living with HIV who are >50 years old in order to reduce the risk of early mortality. This analysis adds to the evolving literature around aging and HIV and risk factors for mortality.

**General comments**:

1) Both male sex and increased years of uncontrolled viral load (low nadir CD4/high WHO stages) are independent risk factors for cardiovascular disease, and a high incidence rate of hypertension in this cohort, especially among female patients, is a notable mortality risk. However, the absence of cause of death data does not allow for further analysis and may reflect limited life expectancy for all adults in Zimbabwe. This should be more clearly indicated as a limitation of the analyses.
2) Given the long duration of 14 years over which this cohort was accrued, the authors should discuss the limitations of temporal trends that could have impacted mortality risks between 2004 and 2020.
3) Additional analyses may further illuminate the high mortality rate, especially since univariate analysis was not impacted by prevalent hypertension. In addition, differences by sex should be explored further by adjusting for CD4 count at treatment initiation. Longitudinal multivariate analysis of incidence hypertension, chronic kidney disease, malignancy, and TB would be needed to support the conclusions of this manuscript.

**Specific comments**:

1) Line 57 –Be more specific about special considerations for HIV care in aging adults living with HIV. Additionally, increased risk of opportunistic infections may not be true in the setting of viral suppression; please provide a reference and modify this comment as needed.
2) Lines 58-59 – Your data and the reference above indicate high rates of adherence in this demographic. Would you consider drug interactions altering drug availability or frequent changes in ART regimen to avoid drug interactions leading to disruptions more accurate that poor adherence?
3) Line 80- do you mean PLHIV (rather than OPLHIV) or does this clinic only provide care to patients over the age of 50 years old?
4) Line 84 – rather than using the term “vulnerable communities,” which implies weakness, UNAIDS terminology guidelines suggest using specific language such as “communities with high prevalence of poverty and unemployment.”
5) Line 132 – Clarify elderly, e.g., “420 patients 50 years or older…”
6) Line 152 – Given the elevated p-value, it would be appropriate to state that males and females died at a similar rate.
7) Line 202- If possible please clarify “cancer of the eye” or replace with “ocular malignancy not specified”
8) Risk of death analysis and Table 4
a) The baseline CD4 count was removed from the multivariate analysis because of collinearity with WHO stage. I would suggest removing WHO stage which is a more subjective measure than a CD4 count, especially given the apparent difference in CD4 count at initiation by sex (Figure 1) in the last decade.
b) Most participants provide multiple measures of blood pressure over time and the incidence of hypertension is quite high. It would be helpful to indicate what proportion of persons reported to have HTN were noted to have elevated BP at multiple visits.
c) Incidence of CKD, TB, and malignancy were also quite high. A longitudinal multivariate analysis of incident hypertension, CKD, TB, and malignancy correlation with mortality risk would be informative.
9) Lines 235-237, 251 – The authors note several differences between sexes in prevalence of social hardship and hypertension with females disproportionately impacted; however, male patients had a higher mortality rate. This warrants a comment from the authors.
10) Line 248 – Please clarify if you are referring to differences between males and females, sex, vs. social roles, genders. The word gender should be replaced with sex in this context.
11) Line 262 – Is this an observation from this cohort? Please ensure that these data on difference in baseline CD4 count and delayed treatment by sex are included in your results section. If these data are not available for your cohort, the reference to Western Cape cohort should be limited to the introduction section.
12) Line 269 – It is not possible to know if patients who died would have stayed in care if they were still living, and thus your data do not support your claim that older patients are more likely to remain committed to care than younger people, especially when the amount of patient tracking and staff outreach may differ at these different clinics.
13) Line 280 – Please comment on the prevalence of peripheral neuropathy and other side effects experienced in your cohort compared to other, younger cohorts.
14) Line 301-303 – Please explain your recommendation for enhancing enrollment strategies for males as a mechanism for reducing mortality among men living with HIV. It is curious that males had high rates of viral suppression but also had a high mortality rate. Figure 1 appears to show lower CD4 at initiation of ART, especially after 2011. Do you have additional data to explain higher mortality rate among males? Can risk of mortality among males be explained by CD4 count at initiation of ART?
15) Line 303-304 – Did unmarried patients have higher likelihood of failing treatment or uncontrolled viral loads? Please provide further data to explain the correlation between unmarried status and mortality in your cohort.
16) Line 320 – Please list all independent predictors of mortality not just WHO stage and unemployment.
17) Conclusion. The results mention the impact of TB and CKD but these were not included in your multivariate (or univariate) analyses. Were these predictors of mortality?
