# Supplementary material for: Treatment outcomes in HIV infected patients older than 50 years attending an HIV clinic in Harare, Zimbabwe: A cohort study
Source: PLoS One. 2021 Jun 9;16(6):e0253000. doi: 10.1371/journal.pone.0253000 (PMC8189507; doi:10.1371/journal.pone.0253000)
Supplement: Attachment — Submitted filename: Response to Reviewers.docx [file pone.0253000.s003.docx]

**Response to Reviewers**

**Responses to the Editor**

1. Please ensure that your manuscript meets PLOS ONE's style requirements, including those for file naming. The PLOS ONE style templates can be found at

<https://journals.plos.org/plosone/s/file?id=wjVg/PLOSOne_formatting_sample_main_body.pdf> and

[https://journals.plos.org/plosone/s/file?id=ba62/PLOSOne_formatting_sample_title_authors_affiliations.pdf](about:blank)

**Response**

We have ensured that our manuscript is consistent with PLOS ONE’s requirements.

2. Please review your reference list to ensure that it is complete and correct. If you have cited papers that have been retracted, please include the rationale for doing so in the manuscript text, or remove these references and replace them with relevant current references. Any changes to the reference list should be mentioned in the rebuttal letter that accompanies your revised manuscript. If you need to cite a retracted article, indicate the article’s retracted status in the References list and also include a citation and full reference for the retraction notice.

**Response**

We have reviewed the reference list and have not found any retracted papers. We have also checked completeness of the references.

3. As part of your revision, please complete and submit a copy of the RECORD checklist, a document that aims to improve reporting and reproducibility of observational studies that use routinely-collected data for purposes of post-publication data analysis and reproducibility: (<http://record-statement.org>). Please include your completed checklist as a Supporting Information file. Note that if your paper is accepted for publication, this checklist will be published as part of your article.

**Response**

Thank you for this recommendation. We have uploaded a copy of the RECORD checklist in this re-submission.

4. We note that you have stated that you will provide repository information for your data at acceptance. Should your manuscript be accepted for publication, we will hold it until you provide the relevant accession numbers or DOIs necessary to access your data. If you wish to make changes to your Data Availability statement, please describe these changes in your cover letter and we will update your Data Availability statement to reflect the information you provide.

Response

The data are currently stored at figshare.com available on the link <https://doi.org/10.6084/m9.figshare.14525487>.

**Reviewer 1**

1) For the definition of hypertension, on "two separate occasions" likely means two different days, as opposed to two measurements on the same day, but recommend clarification.

**Response**

Thank you for this comment. We have reviewed the sentence to read “Diagnosis of hypertension was made when a patient’s systolic or diastolic blood pressure was above 140mm Hg or above 90mm Hg, respectively, on two or more days”

2) Recommend CD4 T cell count to CD4 cell count terminology.

**Response**

We have replaced “CD4 cell count” with CD4 T-cell count.

3) In the abstract, it appears that the number of men and women do not sum to the total studied, but then it becomes clear in the results that the numbers used in the abstract were just referring to the number with a given marital status. Consider showing the denominator of total n men and women in the abstract or another method to clarify.

**Response**

Thank you for noting the discrepancy. We have corrected the figures in both the abstract and Table 1. We have added the denominator in the abstract for clarity.

4) In the introduction, it is stated that there is no similar data on OPLHIV in Zimbabwe - here would be a good place to comment briefly on the existence of OPLHIV data elsewhere in SSA, which is addressed in more detail in the discussion.

**Response**

Thank you for this comment. We have added a comment on data from the region.

5) Recommend highlighting which were the adverse effects attributable to tenofovir, as this is a component the new standard first line regimen, TLD.

**Response**

In the absence of definitive diagnoses of renal impairment which often require kidney biopsies, we could not objectively attribute specific adverse effects to TDF. However, as part of routine clinical care, TDF was stopped with presumptive diagnosis of TDF adverse effects.

6) Would the study have captured aspirin use for risk reduction in those with HTN? Worth a comment, in my view.

**Response**
We have added the statement “Risk reduction of major adverse cardiovascular events using aspirin was only provided for patients with history of myocardial infarction”. These patients are very few.

7) The finding associated with increased risk of mortality and being unmarried merits some additional discussion.

**Response**

We have added the statement “Older patients with HIV face a number of challenges that may affect their quality of life. Loss of partners and friends may lead to social isolation, depression and poor adherence to medicines”.

**Reviewer 2**

**General comments:**

1. Both male sex and increased years of uncontrolled viral load (low nadir CD4/high WHO stages) are independent risk factors for cardiovascular disease, and a high incidence rate of hypertension in this cohort, especially among female patients, is a notable mortality risk. However, the absence of cause of death data does not allow for further analysis and may reflect limited life expectancy for all adults in Zimbabwe. This should be more clearly indicated as a limitation of the analyses.

**Response**

Thank you for this comment. We have added this limitation in the paragraph discussing strengths and weaknesses of the study as highlighted below:

“Cause of death information was not available in the records of deceased patients presenting a limitation in our analysis of risk factors of mortality. Furthermore, our study spans a 16-year period during which mortality risk factors may have evolved significantly.”

2. Given the long duration of 14 years over which this cohort was accrued, the authors should discuss the limitations of temporal trends that could have impacted mortality risks between 2004 and 2020.

**Response**

Thank you for this comment. We have also added this limitation in the paragraph discussing strengths and weaknesses of the study as highlighted above.

3. Additional analyses may further illuminate the high mortality rate, especially since univariate analysis was not impacted by prevalent hypertension. In addition, differences by sex should be explored further by adjusting for CD4 count at treatment initiation. Longitudinal multivariate analysis of incidence hypertension, chronic kidney disease, malignancy, and TB would be needed to support the conclusions of this manuscript.

**Response**

Thank you for this comment. We analysed the mortality differences by sex adjusting for CD4 count alone and the difference was not apparent. However, adjusting for the other confounders showed that indeed sex is an independent risk factor. Instead of performing additional multivariable analyses on hypertension incidence, CKD, malignancy, and TB, we reviewed the conclusion to reflect findings that were observed in the original analysis. The conclusion now reads as follows:

“Our study showed high HIV treatment success among OPLHIV, but with a high burden of hypertension, particularly among women. Low CD4 T-cell counts, male sex, being unmarried and being unemployed were independent predictors of mortality, highlighting the importance of social and socioeconomic factors in addition to the well described early ART for longevity among OPLHIV. We recommend psychosocial support programs to augment the “treat-all” approach aimed at providing ART early before severe immune suppression to improve longevity among OPLHIV.”

**Specific comments:**

1. Line 57 –Be more specific about special considerations for HIV care in aging adults living with HIV. Additionally, increased risk of opportunistic infections may not be true in the setting of viral suppression; please provide a reference and modify this comment as needed.

**Response**

Thank you for this comment. We have reviewed the sentence as follows, “. Management of OPLHIV requires a multidisciplinary team approach, taking into account the onset of non-communicable diseases, premature ageing, and an increased risk of drug-to-drug interactions and drug toxicities due to polypharmacy”.

2. Lines 58-59 – Your data and the reference above indicate high rates of adherence in this demographic. Would you consider drug interactions altering drug availability or frequent changes in ART regimen to avoid drug interactions leading to disruptions more accurate that poor adherence?

**Response**

We agree with your comment. We have reconsidered and edited the sentence as follows: “Polypharmacy also complicates selection of ART regimens and the increased chronic pill burden may lead to treatment fatigue”.

3. Line 80- do you mean PLHIV (rather than OPLHIV) or does this clinic only provide care to patients over the age of 50 years old?

**Response**

Thank you for noting this error. We have corrected the sentence as follows:

“Apart from providing antiretroviral therapy to people living with HIV, the clinic also provides, among other services, onsite laboratory investigations, psychosocial support, reproductive health services including cervical cancer screening, dental services, food support, and bus fare assistance”.

4. Line 84 – rather than using the term “vulnerable communities,” which implies weakness, UNAIDS terminology guidelines suggest using specific language such as “communities with high prevalence of poverty and unemployment.”

**Response**

We have replaced “vulnerable communities” with the suggested terminology.

5. Line 132 – Clarify elderly, e.g., “420 patients 50 years or older…”

**Response**

We have removed the term “elderly” from this sentence and it now reads, “During the study period, 420 patients aged 50 years or older were enrolled into care at Newlands Clinic, 241 (57.4%) being female”

6. Line 152 – Given the elevated p-value, it would be appropriate to state that males and females died at a similar rate.

**Response**

Thank you for this observation. The sentence now reads, “There was no difference in proportion of males died (23.5%) compared to females (19.1%) (p = 0.276)”.

7. Line 202- If possible please clarify “cancer of the eye” or replace with “ocular malignancy not specified”

**Response**

Thank you for the suggestion. We have changed the term as suggested i.e., “ocular malignancy not specified”.

8. Risk of death analysis and Table 4
   a) The baseline CD4 count was removed from the multivariate analysis because of collinearity with WHO stage. I would suggest removing WHO stage which is a more subjective measure than a CD4 count, especially given the apparent difference in CD4 count at initiation by sex (Figure 1) in the last decade.

**Response**

We have repeated the analysis with CD4 included and WHO stage excluded from the Cox model. Table 4 is now updated with new results.

b) Most participants provide multiple measures of blood pressure over time and the incidence of hypertension is quite high. It would be helpful to indicate what proportion of persons reported to have HTN were noted to have elevated BP at multiple visits.

**Response**

We agree that uncontrolled hypertension among hypertensive patients is interesting but would be better addressed as a different study. In the current study, we used the diagnosis variable to determine the burden of hypertension but we did not abstract individual blood pressure measurements from the database. As described in the methods section, a diagnosis of hypertension was made at the clinic when a patient’s systolic or diastolic blood pressure was above 140mm Hg or above 90mm Hg, respectively, on two or more days.

c) Incidence of CKD, TB, and malignancy were also quite high. A longitudinal multivariate analysis of incident hypertension, CKD, TB, and malignancy correlation with mortality risk would be informative.

**Response**

The authors agree with this comment. However, we feel that in-depth analysis of the various aspects listed above would go beyond the scope of this current study and may be better done as separate studies.

9. Lines 235-237, 251 – The authors note several differences between sexes in prevalence of social hardship and hypertension with females disproportionately impacted; however, male patients had a higher mortality rate. This warrants a comment from the authors.

**Response**

We have added the following statement in the discussion: “Despite women being disproportionately affected by adverse social factors as well as hypertension, male patients still had a higher mortality risk in keeping with global general population trends”.

10. Line 248 – Please clarify if you are referring to differences between males and females, sex, vs. social roles, genders. The word gender should be replaced with sex in this context.

**Response**

We have removed the word “gender” from the sentence, and it now reads, “This difference is common in sub-Saharan Africa, where widows are less likely to remarry than widowers.”

11. Line 262 – Is this an observation from this cohort? Please ensure that these data on difference in baseline CD4 count and delayed treatment by sex are included in your results section. If these data are not available for your cohort, the reference to Western Cape cohort should be limited to the introduction section.

**Response**

We agree with your comment. We have removed this sentence.

12. Line 269 – It is not possible to know if patients who died would have stayed in care if they were still living, and thus your data do not support your claim that older patients are more likely to remain committed to care than younger people, especially when the amount of patient tracking and staff outreach may differ at these different clinics.

**Response**

Thank you for this comment. We have edited the sentence to read as, “However, unlike with this larger Zimbabwean study of younger patients wherein 98% of attrition was due to loss to follow-up, 74% of attrition in our cohort was due to death.”

13. Line 280 – Please comment on the prevalence of peripheral neuropathy and other side effects experienced in your cohort compared to other, younger cohorts.

**Response**

We have added the following sentence in the discussion, “Older age has been shown to be associated with peripheral neuropathy in both HIV infected and uninfected people”.

14. Line 301-303 – Please explain your recommendation for enhancing enrollment strategies for males as a mechanism for reducing mortality among men living with HIV. It is curious that males had high rates of viral suppression but also had a high mortality rate. Figure 1 appears to show lower CD4 at initiation of ART, especially after 2011. Do you have additional data to explain higher mortality rate among males? Can risk of mortality among males be explained by CD4 count at initiation of ART?

**Response**

We have added Figure 4 which is depicts Kaplan-Meier survival estimates of mortality by baseline CD4 T-cell counts adjusted for sex, employment, and marital status. From the observed curves, we have edited the comment in the discussion as follows: “The observed trend of persistently higher mortality throughout the follow-up period among people who initiated ART with lower CD4 T-cell counts and the lower baseline CD4 T-cell counts among men affirms the need for a shift in the HIV treatment strategy to improve the ART outcomes observed in men starting with enhanced enrolment strategies”.

15. Line 303-304 – Did unmarried patients have higher likelihood of failing treatment or uncontrolled viral loads? Please provide further data to explain the correlation between unmarried status and mortality in your cohort.

**Response**

This comment has been partly addressed in comment number 7 under Reviewer 1. In addition, we have added the following statements after Table 4:

“Increasing baseline CD4 T-cell counts were associated with lower likelihood of failing ART (p = 0.017), whereas being male, unemployed, or unmarried were not associated with failing ART. Among patients still in care, the number of patients with unsuppressed viral loads was too low to assess for association between independent risk factors of death and having unsuppressed viral loads.”

16. Line 320 – Please list all independent predictors of mortality not just WHO stage and unemployment.

**Response**

Thank you for the comment. We have added all independent predictors of mortality to the conclusion.

17. Conclusion. The results mention the impact of TB and CKD but these were not included in your multivariate (or univariate) analyses. Were these predictors of mortality?

**Response**

Thank you for this comment. The conclusion has been reviewed as shown in the response to comment 3 under general comments.
